# The wounded self—lonely in a crowd: A qualitative study of the voices of children living with atopic dermatitis in Hong Kong

**DOI:** 10.1111/hsc.12917

**Published:** 2019-12-12

**Authors:** Qian‐Wen Xie, Cecilia Lai‐wai Chan, Celia Hoi‐yan Chan

**Affiliations:** ^1^ School of Public Affairs Zhejiang University Hangzhou Zhejiang China; ^2^ Department of Social Work and Social Administration The University of Hong Kong Hong Kong Hong Kong

**Keywords:** atopic dermatitis, family, lived experiences, neighbourhood, qualitative research, school‐aged children, self‐esteem

## Abstract

Atopic dermatitis (AD) imposes significant physical and psychosocial burdens on affected children. However, little has been done to learn from the subjective experiences, perspectives and emotions of children living with AD. Their voices are not heard in healthcare settings. This study aims to share these children's voices and provide a deep understanding of the subjective experiences of children living with AD. We conducted qualitative research by conducting semi‐structured interviews and analysing the drawings of 17 children in Hong Kong aged between 8 and 12 years who were diagnosed with AD. Using a phenomenological approach, we transcribed, coded and described the interviews. We found that for the children in this study, living with AD meant contending with an accumulation of challenges and crises. At the individual level, the essential experience of living with AD manifested a vicious cycle of skin and mental issues. At the family level, conflicts between children and parents concerning AD management coexisted with parental support. The children commonly experienced bullying and isolation in school and discrimination and stigmatisation in their neighbourhood, thereby making living with AD a traumatic experience. The synergy between individual and environmental factors contributed to shaping an incapable and wounded “self” living with AD. Based on our findings, we propose a child‐centred biopsychosocial framework for understanding the living experiences of children with AD. This study suggests different practice strategies for healthcare professionals working with the individual challenges experienced by children living with AD and the challenges these children experience in their family, school, and neighbourhood. The needs of these children should be addressed through an integrated, holistic approach for improving their long‐term health outcomes.

**What is known about this topic:**

- Atopic dermatitis (AD) imposes significant physical and psychosocial burdens on affected children;
- The voices of children living with AD are not heard in healthcare settings;
- Little has been done to learn from the experiences, perspectives and emotions of children living with AD.

**What this study adds:**

- This study provides a deep understanding of the subjective experiences of children living with AD;
- A child‐centred biopsychosocial framework for understanding the lived experiences of children with AD is proposed.

## 1 INTRODUCTION

Atopic dermatitis (AD) is an itchy, non‐infective inflammatory skin disease affecting 15%–30% children and adolescents worldwide (Archer, 2013). AD begins mostly in early childhood; consequently, AD imposes significant physical and psychosocial burdens on children during a critical period of development (Bronkhorst, Schellack, & Motswaledi, 2016). Sleep disturbances affect most children living with AD (Camfferman, Kennedy, Gold, Simpson, & Lushington, 2013). Children whose skins are disfigured by AD often experience discrimination or stigmatisation (Chernyshov, 2016). Previous research has suggested that children living with AD have behavioural problems and experience psychological disturbances, especially psychological stress (Barilla, Felix, & Jorizzo, 2017), anxiety, depression (Cheng et al., 2015) and attention deficit hyperactivity disorder (ADHD; Lee et al., 2016). Generally, children living with AD have a significantly lower quality of life than their healthy peers (Lifschitz, 2015). The higher risk of suicide among the AD population has also received increasing attention in recent years (Picardi, Lega, & Tarolla, 2013).

The United Nations Convention on the Rights of the Child (United Nations, 1989) has helped further emphasise the rights of children and the value of their voices concerning issues that are relevant to them (Harcourt & Einarsdóttir, 2011). Greater attention has been given to child‐friendly practices in the paediatric healthcare system in recent years (Ford, 2011). Much research advocates that information should be sought directly from children with health issues to provide the best appropriate care for them (Ångström‐Brännström & Norberg, 2014; Pickering, Horrocks, Visser, & Todd, 2015).

However, the voices of children living with AD are often not heard by healthcare providers. Secondary evidence is commonly obtained from parents or caregivers, while information is rarely sought directly from the children themselves. Little has been done to learn from the subjective experiences, perspectives and emotions of children living with AD. Furthermore, little is known about how these children connect with their environment, such as their family, school and neighbourhood. How sociocultural factors contribute to shaping the experiences of these children remains unclear.

This research was informed mainly by a biopsychosocial model of disease (Engel, 1977, 1980), which emphasises the impact of the synergy of biological, psychological and social factors on the health outcomes of patients (Wade & Halligan, 2017).

## 2 METHODS

This qualitative study aims to amplify the voices of children living with AD to promote understanding of their subjective experiences. We adapt a phenomenological approach (Moustakas, 1994), which is widely used to study the common meanings of people's lived experiences in specific contexts (Creswell & Poth, 2018).

### 2.1 Participants and sampling

We recruited participants from a group of children (*n* = 55) who were waiting to participate in a psychosocial program specifically for children living with AD in Hong Kong. Only primary schoolers clinically diagnosed as having AD were eligible to participate in this program. By using the SCORing Atopic Dermatitis (SCORAD) index (European Task Force on Atopic Dermatitis, 1993), the assessments of children's severity of AD were administered by the first author, who was well‐trained by a dermatologist in conducting the assessments. The assessments were conducted between June and September 2017. The sociodemographic characteristics of the children were reported by their parents in a questionnaire.

To achieve the maximum variation (in age, gender and severity of AD), we purposely selected a heterogeneous group of 17 children living with AD (eight girls and nine boys aged between 8 and 12 years) to participate in our study (Table 1). Eleven had severe AD, with a SCORAD score above 50 (maximum total score = 103). The remaining six had moderate AD, with a SCORAD score between 25 and 50. Ten participants had shown AD symptoms in the first year of life. Seven had endured AD symptoms for more than 10 years. The six most common types of treatments received by the participants were ointment (*n* = 17), partial steroid treatments (*n* = 17), traditional Chinese medicine (*n* = 16), partial non‐steroid treatments (*n* = 14), wet wrap therapy (*n* = 8) and oral antibiotics (*n* = 8). Five children had other health conditions, such as allergic rhinitis (F6 and M4), asthma (M7), ADHD (M3) and ASD (M5). Two participants (M4 and M6) were from low‐income families with a monthly household income of less than HK$20,000. Fourteen families had more than one child. Nine families had more than one affected child.

**Table 1:** Characteristics of participating children

ID	Gender	Age (y)	Total SCORAD score	Severity of AD	Age at diagnosis (m)	Treatments received	Other conditions	Monthly family income (HKD)	Number of children at home	Number of children with eczema	Interview duration (min)
F1	Female	9	78	Severe	6	123,456	/	NR	2	2	75
F2	Female	12	94	Severe	3	12,356	/	30,000–39,999	2	2	57
F3	Female	8	71	Severe	1	12,356	/	60,000–69,999	2	1	65
F4	Female	10	40	Moderate	0	123,456	/	≥80,000	2	2	62
F5	Female	9	25	Moderate	36	123,456	/	≥80,000	1	1	66
F6	Female	10	63	Severe	48	1,236	AR	NR	4	3	22
F7	Female	9	77	Severe	1	1,236	/	≥80,000	1	1	60
F8	Female	11	82	Severe	3	123,456	/	20,000–29,999	2	1	54
M1	Male	12	61	Severe	2	12,356	/	60,000–69,999	2	2	70
M2	Male	12	43	Moderate	20	123,467	/	20,000–29,999	3	1	34
M3	Male	10	74	Severe	84	126	ADHD	NR	2	2	67
M4	Male	8	47	Moderate	82	12	AR	10,000–19,999	2	2	53
M5	Male	9	46	Moderate	12	12,367	ASD	30,000–39,999	2	1	23
M6	Male	11	78	Severe	2	1,234,567	/	10,000–19,999	1	1	66
M7	Male	8	63	Severe	3	1,246	Asthma	20,000–29,999	2	2	47
M8	Male	10	61	Severe	0	12,346	/	60,000–69,999	2	1	36
M9	Male	11	47	Moderate	48	1,236	/	50,000–59,999	3	3	36

Abbreviations: ID: F = Female, M = Male; Treatment: 1 = ointment, 2 = partial medication (steroid), 3 = partial medication (non‐steroid), 4 = wet wrap therapy, 5 = oral antibiotic, 6 = Traditional Chinese Medicine (TCM), 7 = others; Other conditions: ADHD = attention deficit hyperactivity disorder, ASD = autism spectrum disorder, AR = allergic rhinitis; y = year; m = month.

### 2.2 Data collection

Semi‐structured interviews were conducted with 17 children between September 2017 and February 2018. The draw‐and‐explain technique was used in these interviews to increase the amount of information reported by the children and to enhance their interview performance (Günindi, 2015; Wennström, Hallberg, & Bergh, 2008). Before each interview, the child was provided with one A4 sheet of white paper, one A4 sheet of paper with five concentric circles and a set of 12 coloured pencils. In each interview, the child was asked to draw two pictures. For the first picture, the child was asked to draw whatever he or she would like on a blank piece of paper when thinking of his or her AD. Then, the child was asked to explain his or her drawing. For the second picture, the child was given a sheet of paper with five concentric circles in the centre. (S)he was asked to draw himself or herself in the inner circle and to draw people related to him/her in the surrounding circles; (s)he was asked to place those most important or those he/she liked most closest to him or her and to place those less important or those he/she did not like in the outer circles (Mason & Tipper, 2008). Then, the child was asked to explain his/her relationships with these people and the reasons for their specific locations. A set of open‐ended questions was asked when children explained their drawings; such questions included “Could you please tell me more about this drawing?” and “How do you feel about this?” Two pilot interviews were conducted to fine‐tune the interview procedures and the interview protocol.

The interviews were conducted by five interviewers with a professional background in social work or psychology within a room selected in each social service centre. Each interviewer received 3 hr training: a 1‐hr lecture, 1 hr of role play and 1 hr of interview modelling; professional supervision was provided by the first author. All interviews were audio‐recorded. The tape‐recorded interviews lasted from 22 to 75 min (mean duration: 53 min). Interviews were conducted in Chinese, and the transcripts were reviewed for accuracy by the first author.

### 2.3 Data analysis

All Chinese transcripts of the children's verbal expressions during the interviews were organised and analysed using NVivo software (version 12). Moustakas’s (1994) method was used to analyse the phenomenological data. All transcripts were read in their entirety a minimum of three times before analysis to obtain a sense of the whole database. Significant statements—segments of text relevant to the research questions—were identified and listed. Then, the significant statements were grouped into meaning units (codes), which were then classified into themes. In addition, memos were written to extract concepts from particular phrases, individual interviews and reviews in multiple interviews to track the development of ideas or themes (Creswell & Poth, 2018).

“Epoché” is an important concept in phenomenological research, which was attempted during the data analysis. This concept requires researchers to suspend as much as possible their prejudgments regarding the phenomenon under investigation before attempting to explore the experiences of the participants (Tufford & Newman, 2012). The main investigator (the first author) is a licensed social worker and a Ph.D. with several years of experience conducting research with children living with health issues. She had previously worked closely with more than one hundred families with a child diagnosed as having AD. This experience helped her to understand the difficulties faced by the participants. However, this experience might have biased her understanding of the whole picture of the experiences of children living with AD by inhibiting her ability to see the resilience or strength of the participants.

To reduce bias, a research assistant with a master's degree in social work and who had no experience working with children living with AD also performed the data analysis. The research assistant analysed all the transcripts independently by applying a coding framework developed by the first author. The initial interrater reliability was high (*k* = 0.83). Disagreements were resolved through discussions, which led to the creation of a revised codebook (Table 2). Then, the first author reviewed all the transcripts according to the revised codebook. The transcripts cited in this study were translated into English by the first author and reviewed by the other two authors.

**Table 2:** A Codebook

Theme and subtheme	Meaning unit (Code)
Theme A: Challenges and Crisis	
a. Physical challenges	1. Itching and scratching
	2. Sleep disturbance
	3. Unbearable treatments
	4. Visible skin symptoms and disfigurement
	5. Chronic and relapsing nature
	6. Pain
	7. Short stature
	8. Multiple comorbid conditions
b. Psychological challenges	1. Angry or annoyed
	2. Sad
	3. Worried or afraid
	4. Stressful
	5. Embarrassed
	6. Confused
c. Psychosocial challenges	1. Relationships with parents
	2. Relationships with siblings
	3. Relationships with other family members
	4. Relationship with peers
	5. Relationship with teachers
	6. Relationship with others
d. Cognitive challenges	1. Negative image of eczema
	2. Perceptions of living with eczema
	3. Perceptions of others with eczema
	4. Perceptions of self
	5. Perceived discrimination
e. Academic challenges	1. Fear of examination
	2. Pressure in schoolwork
	3. High expectations of parents
g. Daily‐life challenges	1. Impacting sports or play
	2. Avoiding certain food
	3. Touching
	4. Clothing
Theme B: Social Support	
a. Sources of social support	1. Parents
	2. Siblings
	3. Other family members
	4. Peers
	5. Teachers
	6. Others
b. Types of social support	1. Emotional support
	2. Tangible support
	3. Information support
	4. Social interaction support

### 2.4 Ethical considerations

We received ethical approval for the study from The University of Hong Kong Research Ethics Committee (Ref. EA1707024). Participation was completely voluntary. The purpose and procedures of the study and the rights of the participants were explained to all the participants and their parents. Written informed consent was obtained before the interviews.

## 3 Findings

Based on the relationships among the themes and the subthemes, we organised and reported findings as a composite description, including descriptions of “what” participants experienced with respect to the phenomenon of living with AD and of “how” they experienced the phenomenon.

### 3.1 The child: A vicious circle of skin and mind

#### 3.1.1 Irritability from itching and scratching

Most participants complained about intense pruritus, which was their central and common physical challenge. F4 said that her “hands and feet were jumping when itchy”, while scratching was just like “turning on a light” that could not be turned off even when she realised that she was injuring herself. F2 said that her constant scratching hurt her eyes, for which she required surgery. The constant itching and scratching by the participants frequently caused them psychological stress, such as irritability, and impatience.

#### 3.1.2 Sleeplessness and helplessness

Sleep disturbances caused by itchiness at night were a common complaint for most participants. F3 described her suffering when she could not sleep:

Sometimes AD gets worse when I am asleep… I slept on the bed, it was already 1 o'clock or some time…Then, I slept late, later than 2 o'clock… I had suffered for a long time in bed… I can't sleep at night. I feel that sleeping is the most uncomfortable thing… It should be at 3 o'clock… I usually linger in bed in the morning… I don't want to get up because I can't sleep. And then I am afraid I will fall down and die when I get up.

Frequent sleep disturbances wreaked havoc through long‐term attrition, thereby causing chronic stress in the participants. They often felt powerless and helpless.

#### 3.1.3 Frustration about restrictions in daily life

Skin symptoms largely limited participants’ lifestyles, particularly with respect to diet, play and sports. Seven participants indicated that they had to avoid certain foods to prevent exacerbating their skin conditions. Some participants expressed that they felt particularly itchy when sweating during the summer or after participating in sports and felt considerable pain when bathing or swimming. Participants felt frustrated, angry and unhappy because of the restrictions on their activities.

#### 3.1.4 Chronicity and desperation

Many participants complained about the chronic and recurring process of living with AD. Some perceived that AD was a “long‐term memory” in their lives and would be “always like this forever.” The participants expressed their desperation about the chronic and relapsing nature of AD. According to M6, “AD sticks to the skin. It's always there even when you walk around. You have to take it with you.”

#### 3.1.5 A skin–mind connectedness

F1 indicated the connection between her skin and mood: “Sometimes if my skin is good, my mood is better; if my skin is bad, my mood is worse.” Some participants said that they were often short‐tempered, as they found living with AD “very hard” and “very annoying.” Being perpetually in the grips of chronic stress overload caused by AD, in turn, caused or exacerbated participants’ skin conditions. M1 connected his AD to the stresses in his life: “They are all about emotional things, or stressful things… The main source of my AD is stress.” The participants’ experiences manifested an ongoing and vicious circle of skin (physical symptoms) and mind (psychological distress).

### 3.2 The family: A combination of conflicts and support

#### 3.2.1 “Scratch” and “Don't scratch”

Severe itchiness provoked a universal desire to scratch, subsequently leading to an itch–scratch cycle even though participants actually did not want to scratch. What made them more angry and annoyed was that their parents often ordered them to “don't scratch”. When the participants heard this demand, they thought it was “nonsense,” and they felt “very helpless” because they felt they were not being understood. Sometimes, five participants indicated that their parents criticised, blamed, scolded and beat them when they were scratching. Arguments about scratching and demands to “don't scratch” often caused conflicts and negative emotions for the participants. F5 commented:

They (the parents) don't know how itchy I feel. I don't want to scratch either… They continued to use methods and asked me not to scratch… Sometimes scolding me. I felt very unhappy, and I wanted to get back at them… My parents don't know how bad I feel, they only ask me not to scratch.

#### 3.2.2 Coercion and resistance

In terms of AD treatments, many participants complained about the “uncomfortable,” “sticky” and “spicy” feelings of applying ointments. M7 described his experiences of drinking bitter Chinese medicine, which was perceived as “disgusting” and “smelling bad.” F2 expressed her extreme fear of wet wrap therapy. Some participants also refused AD treatments because they believed that using oral or topical corticosteroids caused their short height, for which they experienced discrimination and bullying. Nevertheless, their parents always “forced” them to rub ointments on their skin or to receive various treatments. In fact, participants actually recognised the benefits of applying ointments and taking oral medicines to treat their skin condition. However, various AD treatments were truly unbearable for them, especially when they were forced to use these treatments. Conflicts with parents about treatments made most participants feel even more distressed and annoyed.

#### 3.2.3 High educational stress from attempting to meet parental expectations

**Figure 1:**
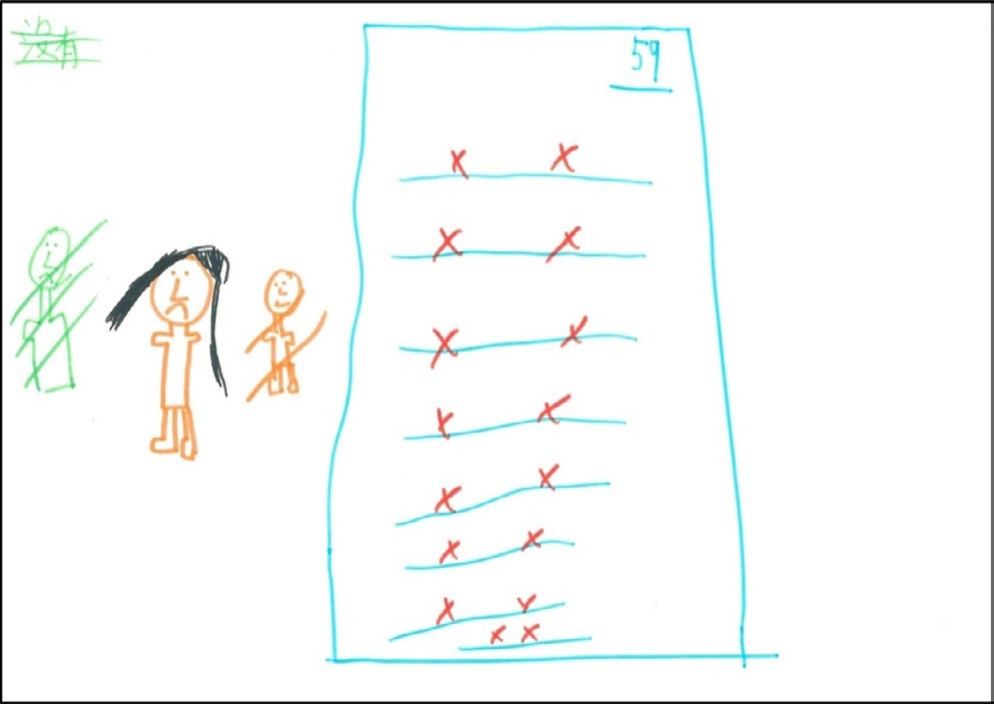
F8’s drawing. F8 drew the result of her last English exam indicating the interaction between AD and her poor academic performance. She also portrayed herself as a lonely girl and crossed out the two people next to her to indicate that she has no friends in school

Nine participants explicitly referred to their educational stress and connected their academic challenges to their AD. The participants believed that their skin became worse when taking tests, as they were under “enormous pressure.” F8 said, “Every time when there is an exam, all AD will come out.” Figure 1 presents her drawing.

More importantly, the participants indicated that their educational stress was mainly due to their parents’ high expectations. As M1 said:

My mother makes high demands on my academic performance. She always wants me to study. It’s stressful.

F3 described her father’ reaction when she wanted to sleep and didn't want to study:

If I cried, then I could sleep. If I said that I wanted to sleep, he still asked me to continue to study… I think my skin would be better if I could sleep more.

Some parents even insisted that good school performance might help to prevent the discrimination children faced as the result of AD. The participants often felt very worried, tense or even guilty when their academic performance did not meet parental expectations.

#### 3.2.4 Love and support amid conflicts

AD caused many conflicts within the family regarding AD management. However, it is important to note that AD might not cause fundamental impairments of the quality of parent–child relationships. Generally, the families of most participants were well functioning and supportive, thereby essentially improving the psychosocial well‐being of the participants. The participants received most of their support—especially tangible and emotional support—from their parents. Parental care, company and encouragement were important resources that helped relieve participants’ stress and anxiety, especially when the participants faced challenges caused by AD.

### 3.3 The school: Full of stress, little support

#### 3.3.1 Peer avoidance or distancing

Participants described their skin symptoms as “red and swollen,” “granular,” “bleeding and oozing,” “full of skin flakes” and “cankered.” Peers’ responses to participants’ skin symptoms and appearance were perceived as major sources of harassment. The participants were disliked, hated and rejected by peers in school due to their AD. Classmates “completely ignored” them and “avoided,” “dodged,” “stepped aside” or “went away” when they saw them, as though their classmates were “seeing a ghost.”

#### 3.3.2 Bullying and victimisation

**Figure 2:**
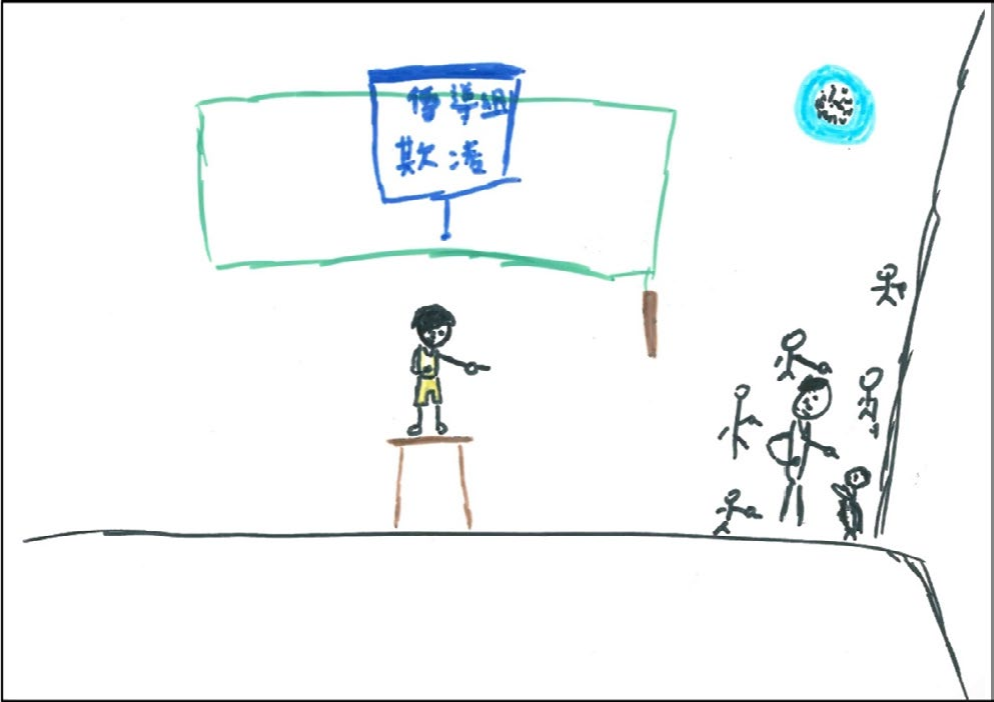
M2’s drawing. M2 portrayed being bullied by his classmates during the lunch break at school. He drew himself kneeling in the lower right corner of picture, surrounded by other students pointing and laughing at him

All participants, except for F6 and M8, described their problematic relationships with peers in school. Moreover, 12 participants indicated that they were bullied verbally, socially and even physically because of their impaired skin. Classmates often laughed at the skin of the participants and even used insulting and demeaning words, such as “virus,” “dirtiness,” “infectious diseases,” “garbage,” “waste” and “ugliness.” M2 depicted being bullied in school in his drawing (Figure 2).

#### 3.3.3 Being disliked by teachers

Participants commonly had very low expectations of their teachers. Some expressed much gratitude and happiness when teachers showed even a little warmth or support, such as saying “hi” to them. However, participants indicated that their teachers often had limited knowledge about AD and negative attitudes towards it. Many participants directly expressed their hatred of their teachers because the latter disliked or ignored them. F2 described her experience of being misunderstood and judged by her teachers due to her skin condition:

They said that I was born with psoriasis and told me not to come to class… A teacher even asked me, “Did you wash your hair? So many flakes.” … I don't want to go to school anymore. I hate the teacher who said that I didn't wash my hair.

#### 3.3.4 A transfer of anger

Most participants exhibited their anger and unhappiness when talking about the avoidance and bullying they experienced in school. They perceived school as a place that gave them chronic stress rather than support. They preferred to endure and put up with these indignities in school but lost their temper at home because they could not see any other escape from the stress. F4 recalled:

Every time when I walked close to a classmate, he stepped away immediately, keeping 1 or 2‐meters distance… I get angry. I am very unhappy. Sometimes I want to cry… I pretended that nothing happened. However, when I got home, I didn't know why I was angry with my mother, very unhappy and angry… In the second year of class, one of my classmates hit me every day, although not very hard, not very painful, but I was very upset… Then, when I got home, I lost my temper every night at home. I didn't like it… I think he hit me because of that (AD).

### 3.4 The neighbourhood: Stigmatisation and discrimination

#### 3.4.1 Being judged by strangers

The participants usually experienced discrimination and stigmatisation in their neighbourhood because strangers often misunderstood their skin disease. F2 described her experience of being accused of having “ADIS” and being called a “leper” by a stranger when she was playing in a public park; the stranger even asked her not to come out so that she would not scare others.

#### 3.4.2 Overreaction and confusion

M1 and M6 said that they felt “very embarrassed” about their skin, especially when other people purposely avoided them. Four participants felt very confused sometimes because they did not understand why other people always “overreacted” to their skin conditions. M6 recalled:

People saw me, and they felt scared many times. I went to the book fair in July. I sat down and read. And then a mother and her daughter came over and saw me. They ran away immediately as if they had seen a ghost… My AD is in fact not infectious. Sometimes I don't understand… I really want normal skin. I don't want to be laughed at.

### 3.5 The wounded self: An outcome of skin, mind and environment

#### 3.5.1 Lonely in a crowd

**Figure 3:**
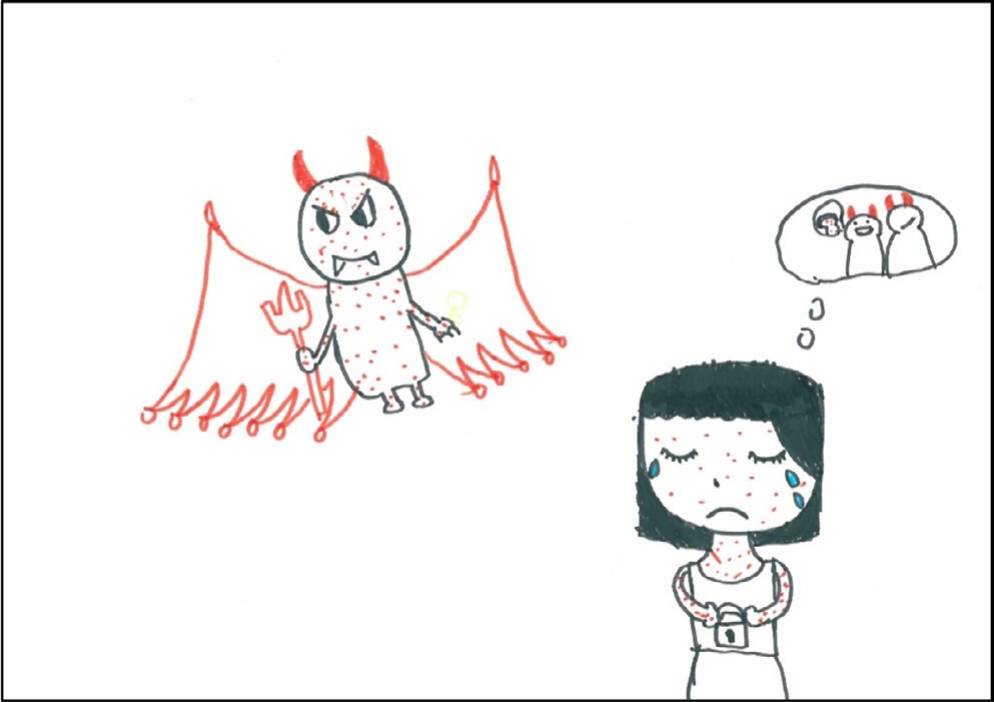
F2’s drawing. F2 drew AD as a red devil with horns and herself as an unhappy girl who was crying. She held a lock in her hands that symbolised interpersonal relationships. However, the AD devil took away the key to this lock. In her mind many people who also looked like devils with horns laughed at her skin

The participants indicated that they felt very lonely because they were not being understood at home and had no friends in school. F2 said that AD “stole the key to my interpersonal relationships.” Her drawing is presented in Figure 3. Deteriorated interpersonal relationships caused their negative self‐image and low self‐esteem, thus aggravating their psychological disturbances.

#### 3.5.2 Self‐stigmatisation and self‐hatred

Several participants thought of themselves as “dirty” and “disgusting” and expressed hatred of their skin. Four participants also said that they wanted to “cover” their skin to prevent it from being seen by others. M6 described:

I don't want my skin to bleed again. I don't want oozing skin anymore… I feel ugly when it was bleeding, I don't want others to see it… So, I often wear long sleeves.

Five participants even thought they were incapable and powerless in other aspects of their lives, such as sports, studying and drawing because they had AD.

#### 3.5.3 Self‐victimisation and self‐denial

Six participants perceived living with AD as “very miserable.” F5 and M1 even thought it was reasonable for classmates and teachers to dislike them because they had AD. They compared themselves unfavourably with other people. F2 said that she wished that she could be an “ordinary person” or even a “foolish person” rather than a person with AD. Five participants thought it was “unfair” that they suffered from AD and “envied others who did not have AD.” As F2 said:

I don't like it (AD). I hate that I have this thing. I feel that God is very unfair. Why do I have this? …. Why do this to me? Why punish me?

Participants repeatedly asked “why me?” and even thought that having AD was a punishment. Desperation was easily found in their descriptions.

### 3.6 A child‐centred biopsychosocial framework

**Figure 4:**
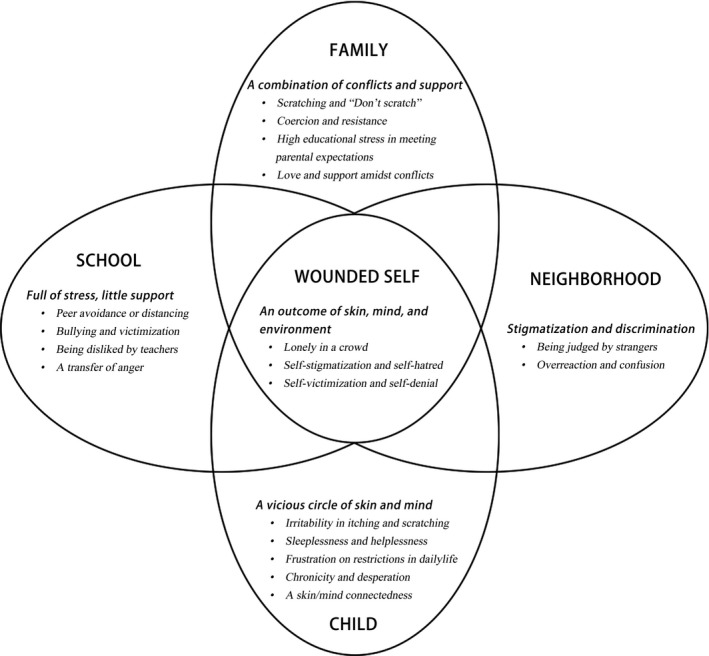
A visual representation of the subjective experiences of participants

The composite description of the experiences of children living with AD contributed to developing a child‐centred biopsychosocial framework (Figure 4). Living with AD means contending not only with the biological symptoms for school‐aged children but also with an accumulation of challenges and crises in their family, school and neighbourhood. The experiences of the affected children were impacted by the synergy of individual and environment factors that subsequently resulted in an incapable and wounded “self.”

## 4 DISCUSSION

The impact of AD on the physical, psychological and social well‐being of children is extensive and pervasive. Nevertheless, children living with AD remain an underrepresented group attracting little social or academic attention. Previous qualitative studies have preferred to focus on the perceptions or experiences of issues related to AD management among the parents and caregivers of children living with AD (Halls et al., 2018; Santer et al., 2015, 2016; Teasdale, Muller, & Santer, 2017). The current qualitative study contributes to amplifying the voices of children living with AD by providing a contextualised description of their subjective experiences.

This study highlights the value of the voices of children living with AD and suggests the capacity of children aged 8–12 years to represent their perspectives and to provide expert testimony about their disease‐related experiences. Consistent with previous studies (Ångström‐Brännström & Norberg, 2014; Einarsdottir, Dockett, & Perry, 2009), this study confirms the appropriateness of an integrated approach combining oral interviews and drawings in qualitative research on the subjective experiences of children with health issues.

By taking the developmental stage of children into account, this study developed a child‐centred biopsychosocial framework for understanding the experiences of children living with AD in a Chinese sociocultural context. At the individual level, the essential experience of these children manifested a vicious circle of skin and mind. Intense itchiness, sleep disturbances, restrictions in daily life and the chronic nature of the disease were perceived as primary challenges, which directly caused emotional distress. The participants’ predominant emotions were anger, annoyance, sadness, unhappiness, worry, fear, embarrassment and confusion. Emotional stress may cause or exacerbate their skin condition, thus creating a vicious circle (Becker‐Haimes, Diaz, Haimes, & Ehrenreich‐May, 2017). Although both our participants and participants composed of young people aged 17–25 years in a recent qualitative study (Ghio et al., 2019) perceived AD as a long‐term and recurring condition, school‐aged children preferred to connect the chronicity of AD to their negative emotions instead of the implications for self‐care. Differences in research objectives and the developmental stages of the participants are possible explanations.

At the family level, conflicts and resources coexisted. Previous qualitative studies found that parents and caregivers employed many strategies to attempt to overcome their children's resistance to AD treatment because they perceived child resistance to be an important barrier to treatment adherence (Santer et al., 2012, 2013). Interestingly, children in this study perceived that their parents’ strategies, especially those involving aggressive actions, strengthened their resistance to treatment. Thus, conflicts between affected children and their parents about AD treatments should be resolved from both sides. Unlike previous studies, which focus on the negative impacts of AD on parent–child relationships from the perspective of parents (Howells et al., 2017; Mitchell, Fraser, Morawska, Ramsbotham, & Yates, 2016), this study emphasises the positive impacts of parental love and support on affected children.

This study also identifies the important influence of high academic stress on the experiences of school‐aged children living with AD. Confucian culture highly values academic excellence (Li, Xue, Wang, & Wang, 2017), as evidenced by the following sentence from an ancient Chinese poem: “Learning (studying) is better than doing any other thing.” High academic stress among children and high parental expectations regarding their children's achievement are very common in Chinese society (Ma, Siu, & Tse, 2018). However, this phenomenon may be more complicated in families that have a child with AD. We found that some parents perceived good school performance as a means of compensating for the limitations imposed by AD on their children. Meanwhile, children living with AD might have a higher risk of experiencing difficulties at school than healthy peers due to their itching skin and sleep disorders (Carroll, Balkrishnan, Feldman, Fleischer, & Manuel, 2005). Educational stress became an important impetus in exacerbating the skin condition of affected children.

Children living with AD commonly experienced bullying and isolation in school and stigmatisation in their neighbourhood; consequently, living with AD became a traumatic experience. Prejudice against those living with AD and fear of being infected may cause peer rejection and social isolation (Ashwanikumar et al., 2018). Smooth, light skin is highly valued in Chinese society and is considered to be a key criterion in defining beauty (Li, Min, & Belk, 2008). Limited knowledge among the general public regarding AD and the perceptions of physical beauty in society may be two fundamental reasons for disapproval of the appearance of children living with AD.

Given their negative experiences in their family, school and neighbourhood, the children in the study often internalised society's standards for beauty and ugliness, thus leading to a negative self‐image and a sense of inferiority. Low self‐esteem may further strengthen the association between their skin conditions and emotional vulnerability (Ashwanikumar et al., 2018).

### 4.1 Implications

Healthcare professionals should target different challenges faced by children living with AD at the individual and environment levels. At the individual level, three important aspects are identified: First, topical therapies for treating physical symptoms should be primary in the management of paediatric AD (Stein & Cifu, 2016). Second, screening procedures for identifying psychological disturbances in children living with AD should be emphasised in conjunction with medical treatment (Bronkhorst et al., 2016). Third, improving the coping skills of children for dealing with teasing and bullying and empowering them to separate their core identity from AD may be useful in helping children living with AD to develop a positive self‐concept and adapt to their environment.

At the family level, resolving conflicts and highlighting resources are equally important. We suggest providing intervention services to children living with AD and their parents together in a parallel format. Contents of intervention programs should integrate educational components (such as those that provide AD‐related information and teach care skills) and psychosocial components (such as those that reduce the psychological stress of both children and parents, improve parenting strategies and improve parent–children relationships).

Working with teachers may be the key to the success of school‐based programs. How teachers treat affected children may not only directly impact the psychological well‐being of these children but also indirectly influence other students’ responses to them. Training teachers about AD, facilitating their awareness of the needs of this group of children, encouraging them to share information on AD with students and improving their skills in recognising and dealing with school bullying may be effective in developing a supportive school environment for children living with AD.

In neighbourhoods, a prevention strategy for attracting social attention to children living with AD and changing public attitudes about AD through education should be the first step.

### 4.2 Limitations and future studies

First, sampling bias might exist in this study. The sample was recruited from a group of children living with AD who registered together with their parents to participate in a psychosocial program in Hong Kong. The participants may be more likely to have a good relationship with their parents and to receive support from them than children whose parents refused to participate in this psychosocial program. A good parent–child relationship may be one reason that AD did not fundamentally impair the parent–child relationships in this sample. More attention should be paid to affected children living in more vulnerable families. In addition, future empirical efforts should also explore the experiences of affected children in different age groups and in other sociocultural contexts. Second, the interviews may be inconsistent because the data were collected by multiple interviewers. Some techniques were used to minimise this limitation and improve the quality of the interviews; these techniques included providing training to all interviewers, using a detailed interview protocol, and involving a supervisor in each interview. Third, the data analysis used in this study was based mainly on children's verbal expressions rather than their drawings. Future research may need to incorporate an analysis of visual data.

## 5 CONCLUSION

Children growing up with AD have a high risk of contending with an accumulation difficulties at the individual and environment levels; it is therefore clear that these children should be treated like a whole person. We suggest that a biopsychosocial focus best addresses the needs of children living with AD through an integrated, holistic approach for improving their long‐term health outcomes.

## CONFLICT OF INTEREST

The authors declared no potential conflicts of interest with respect to the research, authorship and/or publication of this article.

## AUTHOR CONTRIBUTION

Ms. Xie conceptualised and designed the study, analysed data, wrote the initial draft of the manuscript and significantly contributed to revision; Dr. Chan and Prof. Chan critically reviewed and revised the manuscript, and significantly improved manuscript quality; and all authors approved the final manuscript as submitted and agree to be accountable for all aspects of the work.

## References

[hsc12917-bib-0001] Ångström‐Brännström, C. , & Norberg, A. (2014). Children undergoing cancer treatment describe their experiences of comfort in interviews and drawings. Journal of Pediatric Oncology Nursing, 31(3), 135–146. 10.1177/1043454214521693 24651546

[hsc12917-bib-0002] Archer, C. B. (2013). Atopic eczema. Medicine, 41(6), 341–344. 10.1016/j.mpmed.2013.04.002

[hsc12917-bib-0003] Ashwanikumar, B. , Das, S. , Punnoose, V. , Basavaraj, U. , Malathesh, B. , Shoib, S. , & Chatterjee, S. (2018). Interphase between skin, psyche, and society: A narrative review. Indian Journal of Social Psychiatry, 34(2), 99–104. 10.4103/ijsp.ijsp_92_17

[hsc12917-bib-0004] Barilla, S. , Felix, K. , & Jorizzo, J. L. (2017). Stressors in atopic dermatitis. Management of Atopic Dermatitis, 1027, 71–77. 10.1007/978-3-319-64804-0_7 29063432

[hsc12917-bib-0005] Becker‐Haimes, E. M. , Diaz, K. I. , Haimes, B. A. , & Ehrenreich‐May, J. (2017). Anxiety and atopic disease: Comorbidity in a youth mental health setting. Child Psychiatry and Human Development, 48, 528–536. 10.1007/s10578-016-0678-8 27566718

[hsc12917-bib-0006] Bronkhorst, E. , Schellack, N. , & Motswaledi, M. (2016). Effects of childhood atopic eczema on the quality of life: Review article. Current Allergy & Clinical Immunology, 29(1), 18–22. Retrieved from https://www.ingentaconnect.com/content/sabinet/caci/2016/00000029/00000001/art00004.

[hsc12917-bib-0007] Camfferman, D. , Kennedy, D. , Gold, M. , Simpson, C. , & Lushington, K. (2013). Sleep and neurocognitive functioning in children with eczema. International Journal of Psychophysiology, 89, 265–272. 10.1016/j.ijpsycho.2013.01.006 23353660

[hsc12917-bib-0008] Carroll, C. L. , Balkrishnan, R. , Feldman, S. R. , Fleischer, A. B. , & Manuel, J. C. (2005). The burden of atopic dermatitis: Impact on the patient, family, and society. Pediatric Dermatology, 22(3), 192–199. 10.1111/j.1525-1470.2005.22303.x 15916563

[hsc12917-bib-0009] Cheng, C.‐M. , Hsu, J.‐W. , Huang, K.‐L. , Bai, Y.‐M. , Su, T.‐P. , Li, C.‐T. , … Chen, M.‐H. (2015). Risk of developing major depressive disorder and anxiety disorders among adolescents and adults with atopic dermatitis: A nationwide longitudinal study. Journal of Affective Disorders, 178, 60–65. 10.1016/j.jad.2015.02.025 25795537

[hsc12917-bib-0010] Chernyshov, P. V. (2016). Stigmatization and self‐perception in children with atopic dermatitis. Clinical, Cosmetic and Investigational Dermatology, 9, 159–166. 10.2147/CCID.S91263 PMC4959581 27499642

[hsc12917-bib-0011] Creswell, J. W. , & Poth, C. N. (2018). Qualitative inquiry & research design: Choosing among five approaches, 4th ed Thousand Oaks, CA: Sage Publications Inc..

[hsc12917-bib-0012] Einarsdottir, J. , Dockett, S. , & Perry, B. (2009). Making meaning: Children's perspectives expressed through drawings. Early Child Development and Care, 179(2), 217–232. 10.1080/03004430802666999

[hsc12917-bib-0013] Engel, G. L. (1977). The need for a new medical model: A challenge for biomedicine. Science, 196(4286), 129–136. 10.1126/science.847460 847460

[hsc12917-bib-0014] Engel, G. L. (1980). The clinical application of the biopsychosocial model. The American Journal of Psychiatry, 137(5), 535–544. 10.1093/jmp/6.2.101 7369396

[hsc12917-bib-0015] European Task Force on Atopic Dermatitis (1993). Severity scoring of atopic dermatitis: The SCORAD index. Consensus Report of the European Task Force on Atopic Dermatitis. Dermatology, 186, 23–31. 8435513 10.1159/000247298

[hsc12917-bib-0016] Ford, K. (2011). ‘I didn’t really like it, but it sounded exciting’: Admission to hospital for surgery from the perspectives of children. Journal of Child Health Care, 15(4), 250–260. 10.1177/1367493511420185 22089702

[hsc12917-bib-0017] Ghio, D. , Muller, I. , Greenwell, K. , Roberts, A. , McNiven, A. , Langan, S. M. , & Santer, M. (2019). “It’s like the bad guy in a movie who just doesn’t die”: A qualitative exploration of young people’s adaptation to eczema and implications for self‐care. British Journal of Dermatology, 10.1111/bjd.18046 PMC6972719 31021418

[hsc12917-bib-0018] Günindi, Y. (2015). Preschool children's perceptions of the value of affection as seen in their drawings. International Electronic Journal of Elementary Education, 7(3), 371–382. Retrieved from https://www.iejee.com/index.php/IEJEE/article/view/86.

[hsc12917-bib-0019] Halls, A. V. , Nunes, D. , Muller, I. , Angier, E. , Grimshaw, K. , & Santer, M. (2018). ‘Hope you find your ‘eureka’ moment soon’: A qualitative study of parents/ carers’ online discussions around allergy, allergy tests and eczema. British Medical Journal Open, 8(11), 10.1136/bmjopen-2018-022861 PMC6252637 30455386

[hsc12917-bib-0020] Harcourt, D. , & Einarsdóttir, J. (2011). Introducing children's perspectives and participation in research. European Early Childhood Education Research Journal, 19(3), 301–307. 10.1080/1350293X.2011.597962

[hsc12917-bib-0021] Howells, L. M. , Chalmers, J. R. , Cowdell, F. , Ratib, S. , Santer, M. , & Thomas, K. S. (2017). ‘When it goes back to my normal I suppose’: A qualitative study using online focus groups to explore perceptions of ‘control’ among people with eczema and parents of children with eczema in the UK. British Medical Journal Open, 7(11), e017731 10.1136/bmjopen-2017-017731 PMC5695402 29146642

[hsc12917-bib-0022] Lee, C.‐Y. , Chen, M.‐H. , Jeng, M.‐J. , Hsu, J.‐W. , Tsai, S.‐J. , Bai, Y.‐M. , … Su, T.‐P. (2016). Longitudinal association between early atopic dermatitis and subsequent attention‐deficit or autistic disorder A population‐based case‐control study. Medicine, 95(39), 10.1097/%2FMD.0000000000005005 PMC5265954 27684861

[hsc12917-bib-0023] Li, E. P. , Min, H. J. , & Belk, R. W. (2008). Skin lightening and beauty in four Asian cultures. ACR North American Advances. Retrieved from. http://acrwebsite.org/volumes/13415/volumes/v35/NA‐35.

[hsc12917-bib-0024] Li, M. , Xue, H. , Wang, W. , & Wang, Y. (2017). Parental expectations and child screen and academic sedentary behaviors in China. American Journal of Preventive Medicine, 52(5), 680–689. 10.1016/j.amepre.2016.12.006 28108188

[hsc12917-bib-0025] Lifschitz, C. (2015). The impact of atopic dermatitis on quality of life. Annals of Nutrition and Metabolism, 66, 34–40. 10.1159/000370226 25925339

[hsc12917-bib-0026] Ma, Y. , Siu, A. , & Tse, W. S. (2018). The role of high parental expectations in adolescents’ academic performance and depression in Hong Kong. Journal of Family Issues, 39(9), 2505–2522. 10.1177/0192513X18755194

[hsc12917-bib-0027] Mason, J. , & Tipper, B. (2008). Being related: How children define and create kinship. Childhood Education, 15(4), 441–460. 10.1177/%2F0907568208097201

[hsc12917-bib-0028] Mitchell, A. , Fraser, J. , Morawska, A. , Ramsbotham, J. , & Yates, P. (2016). Parenting and childhood atopic dermatitis: A cross‐sectional study of relationships between parenting behaviour, skin care management, and disease severity in young children. International Journal of Nursing Studies, 64, 72–85. org/10.1016/j.ijnurstu.2016.09.016 27693983 10.1016/j.ijnurstu.2016.09.016

[hsc12917-bib-0029] Moustakas, C. E. (1994). Phenomenological research methods. Thousand Oaks, CA: Sage Publications Inc.

[hsc12917-bib-0030] Picardi, A. , Lega, I. , & Tarolla, E. (2013). Suicide risk in skin disorders. Clinics in Dermatology, 31(1), 47–56. 10.1016/j.clindermatol.2011.11.006 23245973

[hsc12917-bib-0031] Pickering, D. M. , Horrocks, L. M. , Visser, K. S. , & Todd, G. L. (2015). Analysing mosaic data by a ‘Wheel of Participation’ to explore physical activities and cycling with children and youth with cerebral palsy. International Journal of Developmental Disabilities, 61(1), 41–48. 10.1179/2047387714Y.0000000038

[hsc12917-bib-0032] Santer, M. , Burgess, H. , Yardley, L. , Ersser, S. , Lewis‐Jones, S. , Muller, I. , … Little, P. (2012). Experiences of carers managing childhood eczema and their views on its treatment: A qualitative study. British Journal of General Practice, 62(597), 261–267. 10.3399/bjgp12X636083 PMC3310032 22520913

[hsc12917-bib-0033] Santer, M. , Burgess, H. , Yardley, L. , Ersser, S. J. , Lewis‐Jones, S. , Muller, I. , … Little, P. (2013). Managing childhood eczema: Qualitative study exploring carers' experiences of barriers and facilitators to treatment adherence. Journal of Advanced Nursing, 69(11), 2493–2501. 10.1111/jan.12133 23528163

[hsc12917-bib-0034] Santer, M. , Muller, I. , Yardley, L. , Burgess, H. , Ersser, S. J. , Lewis‐Jones, S. , & Little, P. (2015). 'You don't know which bits to believe': Qualitative study exploring carers' experiences of seeking information on the internet about childhood eczema. British Medical Journal Open, 5(4), e006339 10.1136/bmjopen-2014-006339 PMC4390694 25854963

[hsc12917-bib-0035] Santer, M. , Muller, I. , Yardley, L. , Lewis‐Jones, S. , Ersser, S. , & Little, P. (2016). Parents’ and carers’ views about emollients for childhood eczema: Qualitative interview study. British Medical Journal Open, 6(e011887), 1–9. 10.1136/bmjopen-2016-011887 PMC5013451 27543590

[hsc12917-bib-0036] Stein, S. L. , & Cifu, A. S. (2016). Management of atopic dermatitis. JAMA, 315(14), 1510–1511. 10.1001/jama.2016.1459 27115267

[hsc12917-bib-0037] Teasdale, E. , Muller, I. , & Santer, M. (2017). Carers’ views of topical‐corticosteroid use in childhood eczema: A qualitative study of online discussion forums. British Journal of Dermatology, 176(6), 1500–1507. 10.1111/bjd.15130 27753076

[hsc12917-bib-0038] Tufford, L. , & Newman, P. (2012). Bracketing in qualitative research. Qualitative Social Work, 11(1), 80–96. 10.1177/%2F1473325010368316

[hsc12917-bib-0039] United States (1989). Convention on the rights of the child. New York: United Nations General Assembly.

[hsc12917-bib-0040] Wade, D. T. , & Halligan, P. W. (2017). The biopsychosocial model of illness: A model whose time has come. Clinical Rehabilitation, 31(8), 995–1004. 10.1177/0269215517709890 28730890

[hsc12917-bib-0041] Wennström, B. , Hallberg, L. R. M. , & Bergh, I. (2008). Use of perioperative dialogues with children undergoing day surgery. Journal of Advanced Nursing, 62(1), 96–106. 10.1111/j.1365-2648.2007.04581.x 18352968

